# Layered LiCoO_2–_LiFeO_2_ Heterostructure Composite for Semiconductor-Based Fuel Cells

**DOI:** 10.3390/nano11051224

**Published:** 2021-05-06

**Authors:** Yanyan Liu, Chen Xia, Baoyuan Wang, Yongfu Tang

**Affiliations:** 1 Hebei Key Laboratory of Applied Chemistry, College of Environmental and Chemical Engineering, Yanshan University, Qinhuangdao 064004, China; tangyongfu@ysu.edu.cn; 2 Faculty of Physics and Electronic Science, Hubei University, Wuhan 430062, China; chenxia@hubu.edu.cn

**Keywords:** heterostructure composite, LiCoO_2_–LiFeO_2_, semiconductor-based fuel cell, high performance

## Abstract

Enabling fast ionic transport at a low-temperature range (400–600 °C) is of great importance to promoting the development of solid oxide fuel cells (SOFCs). In this study, a layer-structured LiCoO_2_–LiFeO_2_ heterostructure composite is explored for the low-temperature (LT) SOFCs. Fuel cell devices with different configurations are fabricated to investigate the multifunction property of LiCoO_2_–LiFeO_2_ heterostructure composites. The LiCoO_2_–LiFeO_2_ composite is employed as a cathode in conventional SOFCs and as a semiconductor membrane layer in semiconductor-based fuel cells (SBFCs). Enhanced ionic conductivity is realized by a composite of LiCoO_2_–LiFeO_2_ and Sm^3+^ doped ceria (SDC) electrolyte in SBFC. All these designed fuel cell devices display high open-circuit voltages (OCVs), along with promising cell performance. An improved power density of 714 mW cm^−2^ is achieved from the new SBFC device, compared to the conventional fuel cell configuration with LiCoO_2_–LiFeO_2_ as the cathode (162 mW cm^−2^ at 550 °C). These findings reveal promising multifunctional layered oxides for developing high-performance LT–SOFCs.

## 1. Introduction

As energy demands continue to increase, solid oxide fuel cell (SOFC) as a high-efficiency and environmentally friendly energy conversion technology has received more and more attention [1,2]. Typically, a SOFC device is constructed of three functional components including a porous cathode–anode and a dense electrolyte layer [3]. The electrolyte mainly functions as a membrane to transfer ions and blocks electrons to avoid an internal short-circuiting issue [4]. Among various electrolyte materials, Y_2_O_3_-doped ZrO_2_ (YSZ) was proved as the best with high ionic conductivity, reaching up to 0.1 S cm^−1^ at 1000 °C [5,6]. However, an elevated operating temperature (e.g., 1000 °C) is essential for YSZ to achieve sufficiently high ionic conduction and realize a good fuel cell performance. The high operating temperature requirement, combined with technical difficulty and high cost, significantly limits the SOFC commercialization [5,7,8].

Recently, semiconductors are selected to serve as novel electrolytes or key components in SOFCs, particularly in a proposed concept of the semiconductor-based fuel cell (SBFC) technology [9,10,11,12,13]. Analogously to the conventional SOFC, the SBFC technology can realize fuel cell function by converting chemical energy into electrical power. Differently, the ion-conducting electrolyte separator is removed instead of an integrated semiconductor–ionic conductor layer. The formation of bulk heterogeneous p–n junction or Schottky junction can avoid the internal short circuit issue in the SBFC devices [9,14]. The semiconductor–ionic conductor layer in SBFC has been dominantly investigated based on the dual- or multiphase homogeneous composites [15,16,17,18,19]. For example, the typical ionic conductors Gd^3+^- or Sm^3+^-doped ceria (GDC or SDC) are composited with a semiconducting LiNiZn-based oxide as the core component to realize the fuel cell functions [10]. In this regard, the ion-conducting and semiconducting phases can provide the percolating passageways for ions’ transfer, including O^2−^ and H^+^ ions, while the electron conduction is suppressed by the formed junction in the semiconductor phase.

Furthermore, Tao et al. reported a single-phase layer-structured semiconductor Li_x_Co_0_._5_Al_0_._5_O_2_, which was employed in an analogous SBFC device and achieved a power density of 150 mW cm^−2^ at 650 °C [20]. As claimed, the layer-structured Li_x_Co_0_._5_Al_0_._5_O_2_ exhibited high ionic conductivity, as 0.1 S cm^−1^ at 500 °C under H_2_–air condition. As known, the transition metal oxides can possess inherent electronic conductivity. Interestingly, the electronic conductivity of this material did not cause any internal short-circuit issue in this device during fuel cell working. Parallelly, another electron-conducting LiFeO_2_ and insulating LiAlO_2_ composite was reported to display a quite high ionic conductivity as 0.24–0.50 S cm^−1^ at the temperature range of 600–650 °C under H_2_–air fuel cell condition [21]. The authors proposed that this layer-structured composite possessed multi-ionic conductivities including Li^+^, H^+^, O^2−^ and even e^-^ in SOFC application. Likewise, no short-circuiting problem was observed in this device.

In this work, we further investigate semiconductor composites based on two layer-structured semiconductors LiCoO_2_ and LiFeO_2_ for SBFC technology. A hybrid triple ion and electron conduction can be expected. Various designed molar ratios of LiCoO_2_ and LiFeO_2_ are prepared to select the optimum one for SBFC. The aforementioned materials are characterized by the material characterization methods, e.g., X-ray diffraction (XRD), scanning electron microscope (SEM), and transmission electron microscopy (TEM). Additionally, an electrochemical impedance spectrum (EIS) is employed here to analyze the electrical property of the LiCoO_2_–LiFeO_2_ heterostructure composite. Furthermore, the electrochemical properties of LiCoO_2_–LiFeO_2_ composite for SBFC are detailly discussed to clarify the mechanism of the enhanced fuel cell performance.

## 2. Materials and Methods

### 2.1. Material Preparation and Characterization

All the chemicals in the analytical grade were purchased from Sinopharm Chemical Reagent Co., Ltd., Wuhan, China. All of them were used without further purification. The LiCoO_2_–LiFeO_2_ heterostructure composite (abbreviated as LCF) was synthesized via a facile hydrothermal method. Typically, 0.02 M lithium acetate was dissolved in deionized water. Then, 0.01 M cobalt nitrate and 0.01 M iron nitrate in the same volume were added, following magnetic stirring. Subsequently ammonium hydroxide with a concentration of 2 M, as precipitator, was dropwise added to pH up to 9–10. After stirring for 2 h, the solution was transferred into the Teflon reactor at 120 °C for 4 h. The precipitate was filtered and washed several times by distilled water, followed by drying at 120 °C to obtain the precursor and then calcined at 720 °C for 2 h to obtain the final LiCoO_2_–LiFeO_2_ composite. To clarify the effect of composition, the LiCoO_2_–LiFeO_2_ composites with various weight ratios of 8:2, 5:5, and 2:8, denoted as LCF2, LCF, and LCF8, respectively, are designed and synthesized in the same procedure.

The preparation process of samarium doped ceria (SDC) has been reported in a previous publication [22]. The commercial nickel cobalt aluminum–lithium oxides (Ni_0_._8_Co_0_._15_Al_0_._05_Li-oxide, NCAL) used in this study, were purchased from Tianjin Bamo Sci.&Tech. Joint Stock Ltd., Tianjin, China.

The structure of the as-synthesized LCF sample was detected by X-ray diffraction (XRD, D-max-2500 X-ray diffractometer, Japanese Rigaku Corp., Tokyo, Japan) with filtered Cu–Kα radiation. The data were recorded in the 2 theta range of 20–90° and analyzed using Jade 6.5 software. The morphology of the LCF sample was characterized on a field emission scanning electron microscopy (FE-SEM, Hitachi S-4800, Tokyo, Japan) equipped with an energy dispersive spectrometer (EDS), transmission electron microscope (Hitachi HF-7700, Tokyo, Japan) and the high-resolution transmission electron microscope (Titan ETEM G2, Hillsboro, OR, USA).

### 2.2. Fuel Cell Fabrication and Electrochemical Measurements

Three types of fuel cells based on the as-synthesized LCF sample were investigated, noted as Cell I, Cell II, and Cell III. In Cell I, LCF2, LCF, and LCF8 were employed, respectively, to select the optimum component for further study. The three samples were composited with SDC in a weight ratio of 4:6, respectively, as the core layer (0.3 g) of the SBFC device (Cell I). In this device, NCAL (0.1 g) and LCF (0.1 g) were attached to the hydrogen input side and air input side, respectively. This type of fuel cell configuration is written as (H_2_-input side) Ni-foam/NCAL | SDC-LCF composite | LCF (air-input side). To clarify the optimum ratio of LCF and SDC further, the LCF sample was composited with SDC in different weight ratios of 2:8, 3:7, and 4:6 denoted as 8SDC/2LCF, 7SDC/3LCF, and 6SDC/4LCF. These composites were used in the same configuration of Cell I. Cell II was fabricated into a conventional SOFC configuration. The prepared LCF (0.1 g) was used as the cathode, while the mixed NCAL and SDC powders with a volume ratio of 2:3 (0.3 g) were employed as the anode, and foam nickel was attached as a current collector. Additionally, SDC (0.1 g) was used as an electrolyte. The configuration of Cell II can be described as Ni-foam/SDC + NCAL (anode) | SDC (electrolyte) | LCF (cathode). For Cell III, the bare LCF was employed as the core component, while NCAL pasted into nickel foam was attached to both sides of this device. That is (H_2_-input side) Ni-foam/NCAL | LCF | NCAL/Ni-foam (air-input side).

All of the as-fabricated fuel cells in a disc-type green cell with a diameter of 13 mm were co-pressed using a pressure of 150 MPa. These pellets are approximately 1 mm in thickness with an active area of 0.64 cm^2^. A programmable electronic load (ITECH8511, ITECH Electrical Co., Ltd., Nanjing China.) was employed here to measure the fuel cell performances. Hydrogen was used as the fuel with a flow of 120 mL min^−1^ at a pressure of 1 bar. The diagram of testing station is shown in Figure S1.

The electrical conductivity of as-prepared LCF material was fabricated via a dry-pressing process using LCF pellet with silver pastes served as current collectors. The electrochemical impedance spectroscopy (EIS) was conducted on an electrochemical workstation (CHI660B, Chen Hua Corp., Shanghai, China) with operational temperatures from 600 °C to 400 °C in air under an open circuit condition. The experimental data were simulated and analyzed on Zview software (Scribner Associates Inc., Southern Pines, NC, USA). The calculation of electrical conductivity σ can be conducted based on the fitting results using the following equation as σ = L/RA. Here, L is the thickness of the samples, R is the total resistance, and A represents the cross-sectional area.

## 3. Results

### 3.1. Material Characterizations

**Figure 1:**
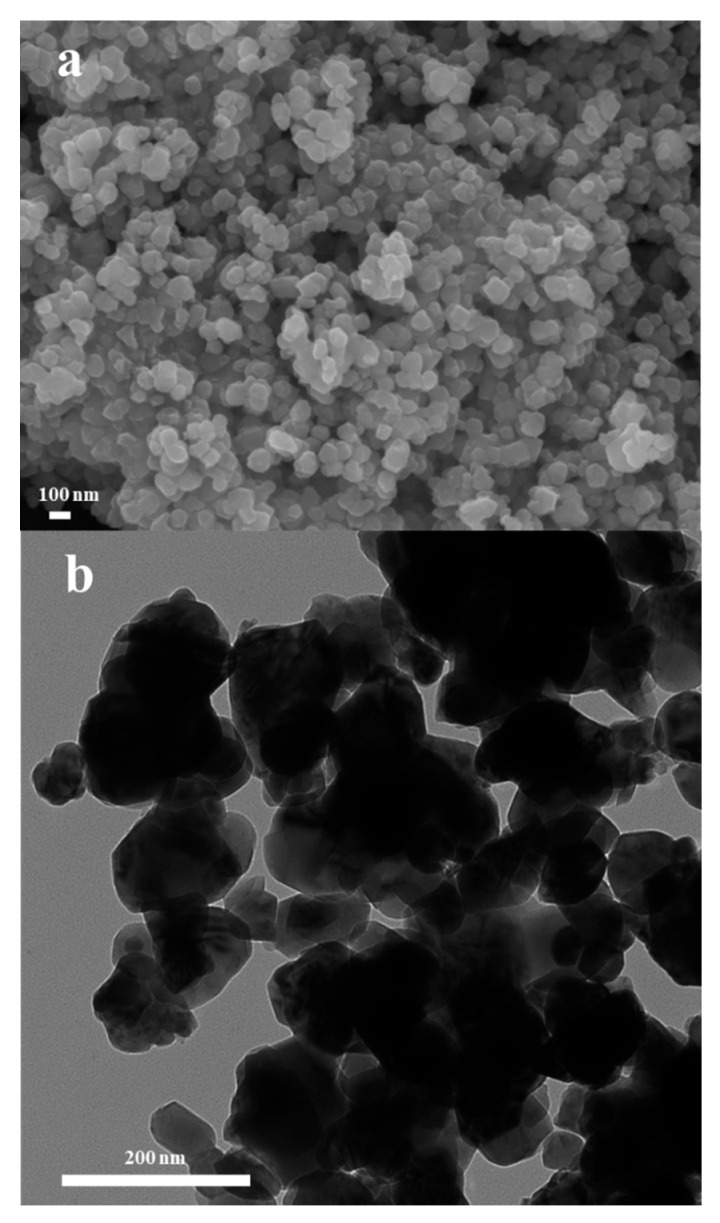
(**a**) SEM and (**b**) TEM micrographs of LCF sample.

**Figure 2:**
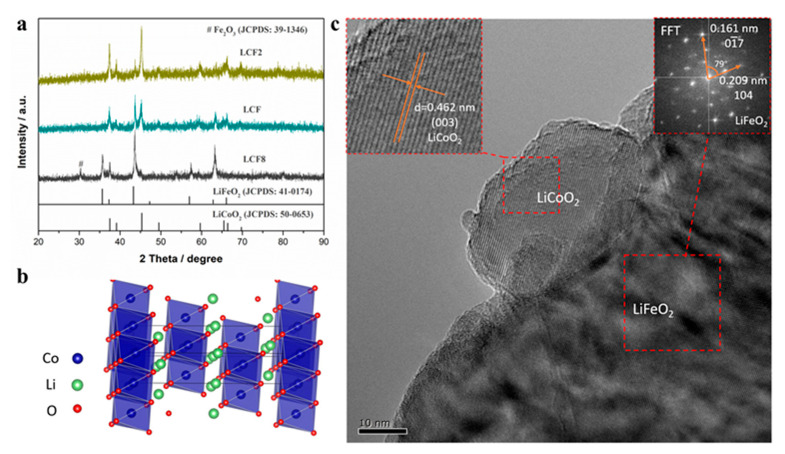
(**a**) XRD patterns for LCF2, LCF, and LCF8 samples synthesized via hydrothermal method. (**b**) Diagram of layer-structured LiCoO_2_. (**c**) HRTEM image of LCF nanocomposite. The enlarged image of the LiCoO_2_ particle is inserted in the top left corner. Additionally, the corresponding fast Fourier transform (FFT) pattern of LiFeO_2_ particle is located in the top right corner.

Figure 1a presents the plane view of scanning electron microscopy (SEM) microstructure of the as-synthesized LCF sample by hydrothermal method. The transmission electron microscopy (TEM) of the LCF sample is shown in Figure 1b. It can be observed that the LCF particles show a homogeneous distribution in a nanometer scale from both SEM and TEM images. The observed particle sizes range approximately between 60 and 90 nm. The relatively small particles facilitate the ionic or electronic conduction and improve the surface activity of LCF. The X-ray diffraction (XRD) patterns for as-synthesized LCF2, LCF, and LCF8 samples are demonstrated in Figure 2a. As observed, the LCF2 and LCF samples have similar diffraction peaks. These peaks can be assigned to the standard patterns of layer-structured LiCoO_2_ (JCPDS No. 50-0653) and LiFeO_2_ (JCPDS No. 41-0174), implying the LiCoO_2_–LiFeO_2_ composites are obtained without reactions occurring between the LCF and LCF2. The different intensities of the diffraction peaks indicate the LiCoO_2_–LiFeO_2_ composites with the different composition ratios in the two samples [23]. In the high Fe-containing LCF8 material, the diffraction peaks correspond to the standard pattern of LiFeO_2_ (JCPDS No. 41-0174) with a slight shift to higher angles, indicating the partial substitution of the Fe ions by the Co ions with a smaller radius. Additionally, the emerging peak at around 30° is consistent with the standard Fe_2_O_3_ patterns (JCPDS No. 39-1346), indicating the LCF8 sample was composed with Co-doped LiFeO_2_ and Fe_2_O_3_. The energy dispersive spectrometer (EDS) spectra and corresponding composition analysis in Figure S2 in Supporting Information confirmed the existence of Co and Fe in a nearly designed stoichiometric ratio of 1:1. In EDS spectra, the Li element is not detected due to its low atomic number. It should be noted that all of the as-synthesized samples are likely to be lithium deficient since the sublimation of Li element during high-temperature sintering process. The layered structure of LiCoO_2_ is demonstrated in Figure 2b. The two-dimensional CoO_2_ layers consisting of edge-sharing CoO_6_ octahedra are separated by lithium layers [20,21]. The HRTEM image in Figure 2c illustrates a LiCoO_2_-LiFeO_2_ nanocomposite structure. The smaller nanoparticle is characterized as LiCoO_2_ featured by the lattice plane distance of 0.462 nm, which fits well with the (003) lattice plane of LiCoO_2_. This LiCoO_2_ nanoparticle is composited with a bigger LiFeO_2_ nanoparticle. In the fast Fourier transform (FFT) pattern from the bigger particle, the diffraction spots corresponding to 0.209 nm and 0.161 nm lattice plane distance are indexed as 104 and $0\bar{1}7$ diffraction spots of LiFeO_2_, respectively. The measured cross angle between (104) and ($0\bar{1}7$) lattice plane is 79 degrees, which is consistent with the reference structure of LiFeO_2_ (JCPDS: 41-0174). Evidenced by the HRTEM image, LiCoO_2_ and LiFeO_2_ nanoparticles are phase separation in the LCF sample, and the clear grain interfaces can be formed between the two phases.

### 3.2. Electrical Properties

**Figure 3:**
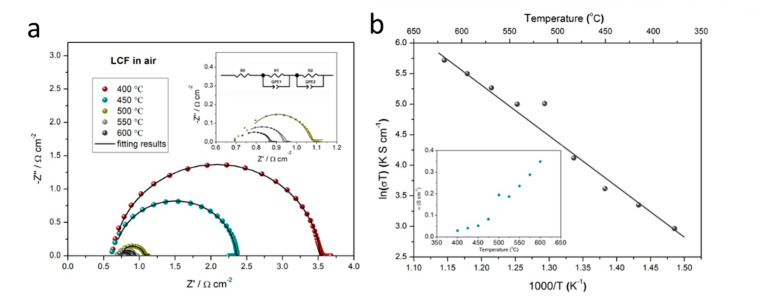
(**a**) Selected EIS patterns for LCF sample measured at various temperatures (400–600 °C in an interval of 25 °C) in air (insert images are the employed equivalent circuit for fitting and the enlarged EIS measured at 550, 550, and 600 °C). (**b**) Temperature-dependence Arrhenius plot in terms of ln(σT) versus 1/T and the corresponding conductivities are plotted in the insert versus temperatures.

To understand the electrochemical kinetics of the layered LCF heterostructure composite, the electrochemical impedance spectroscopy (EIS) was performed in the air atmosphere for clarifying the intrinsic oxygen ion or electron conduction. The Nyquist profiles of LCF at different measured temperatures are presented in Figure 3a. In the qualitative view, the impedance spectra of the LCF sample demonstrate a general mixed conducting property with a high-frequency intercept, a medium frequency semicircle, and a short low-frequency arc [24]. The simulations for the as-measured impedance spectra were conducted by employing Zview software. An empirical equivalent circuit model of a resistor (R_o_) in series with R_1_//QPE_1_ and R_2_//QPE_2_ (a resistor R in parallel with a constant phase element QPE) circuit (as shown in the inset of Figure 3a), was employed to analyze the particular kinetic behaviors in LCF pellet with thin silver paste as current collectors at both sides. As observed, the equivalent model fits the experimental data well. In this model, QPEs generally refer to the constant phase element to reflect the “depressed” arc of impedance spectra, taking into account inhomogeneity in some unideal cases [25,26]. R_o_ is related to the grain contribution from intrinsic oxygen ion transfer in bulk LCF, located at the high-frequency region in the EIS plot. R_1_ is assigned to the diffusion of atomic oxygen at the grain boundary indexing to the medium frequency semicircle. The low-frequency resistance of R_2_ is associated with the electrode polarization process. The calculation of electrical conductivity σ can be conducted based on EIS fitting results using the equation as. Here, L is the thickness of the pellet as around 1 mm, R is the total resistance, and A represents the cross-sectional area of 0.64 cm^−2^. The total conductivities for the LCF sample are estimated as 0.03–0.34 S cm^−1^ in the temperature range of 400–600 °C. The roughly calculated electrical conductivities are plotted as insert images versus different temperatures, and the Arrhenius plots based on total conductivities are presented in Figure 3b. The conductivities are significantly higher than the common oxygen–ion or proton-conducting electrolytes, even higher than some nanocomposite electrolytes [5,27,28,29,30]. The increasing electrical conductivity can be attributed to the existence of electron conduction due to the multivalent Co and Fe ions.

**Figure 4:**
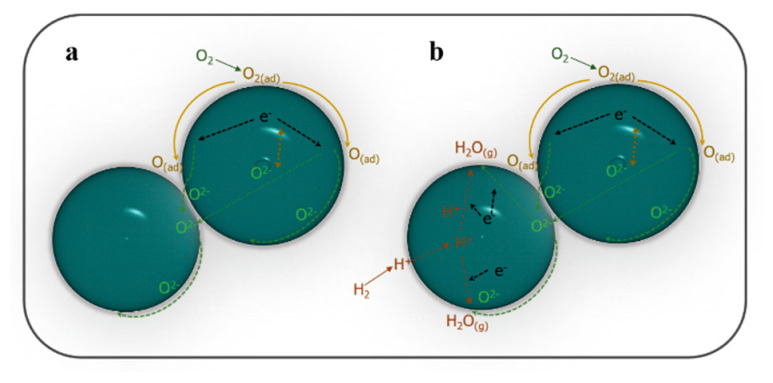
Schematic illustrations of (**a**) oxygen species transport and (**b**) mixed H^+^ and O^2−^ transport enhanced by electron transfer in LCF.

For transition metal oxides, multivalent ions or doping asymmetry can contribute to various electrical properties, e.g., insulating, semiconducting, or metallic, involving different defect types [31,32,33]. It can be observed in Figure 3a that the EIS plots exhibit mixed electron–ion conducting properties with ionic conductivity as the dominant one. Particularly, the oxygen vacancy defects are speculated as a primary factor in this LCF material. As demonstrated, oxygen molecules are primarily absorbed and transported along the surface of particles, and then they are catalyzed and reduced into ionic or atomic oxygen species. Subsequently, these oxygen species are transferred through two pathways, along the surface or cross the bulk, into the neighboring particles. In this process, the transport of oxygen ions might be enhanced by the movements of electrons. It has been proved that the ion conduction can be significantly improved for an ionic conductor by compositing with another phase, e.g., carbonate, semiconductor, even insulating conductor [34,35,36,37]. Similarly, the mixed electron–ion conducting LCF material has synergetic enhanced mobility of ion transfer by electron transport. As reported by Lan and Tao, layer-structured oxides have great potential for proton transport [20]. The proton exchange is expected in the layered LiCoO_2_–LiFeO_2_ composite. Particularly, the interfaces between the LiCoO_2_ and LiFeO_2_ phases possibly enhance the proton transfer. The proposed oxygen and proton species transport pathways in the LiCoO_2_–LiFeO_2_ composite are illustrated in Figure 4.

### 3.3. Electrochemical Performance

**Figure 5:**
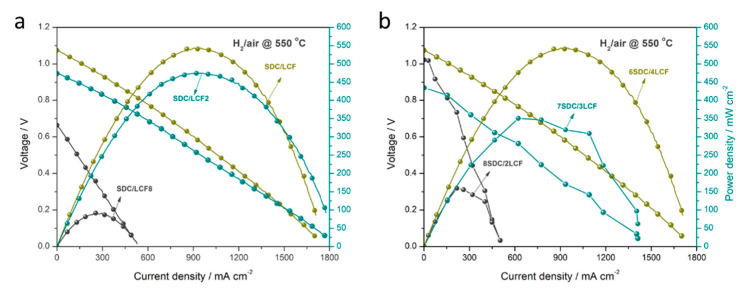
I–V and I–P characteristics of the fuel cell devices (**a**) using SDC/LCF2, SDC/LCF, and SDC/LCF8 composites in a weight ratio of 6:4 and (**b**) using various weight ratios of SDC and LCF composite.

In previous reports, the layer-structured transition metal oxides, e.g., LiNi_0_._1_Fe_0_._9_O_2-δ_ or LiNi_0_._8_Co_0_._2_O_2_ have been used in the developing SBFC technology with excellent fuel cell performances [38,39,40]. Hereby, the other LCF2, LCF, and LCF8 composites are primarily tested using an assembled SBFC device based on SDC/LCF composite. In this regard, LCF samples are expected to improve the electrical conductivity via composited with ionic conductor SDC. As reported, the electronic conducting LiFeO_2_ composited with an insulating γ-LiAlO_2_ exhibited dominating dual ions (O^2−^ and H^+^) conductivity under H_2_–air fuel cell conditions [21]. Analogously, the LiCoO_2_–LiFeO_2_ composite is designed to provide enhanced ionic conduction for SDC. Figure 5a depicts the I–V characteristics and corresponding output power densities for the SBFC devices with electronic–ionic conducting SDC/LCF composites at 550 °C. The different composition LCF2, LCF, and LCF8 samples are performed to identify the optimum one. The fuel cell device using SDC/LCF composite achieves the highest open-circuit voltage (OCV) of 1.07 V and the corresponding best peak power density output of 541 mW cm^−2^ at 550 °C, while both the tablet devices using the SDC/LCF2 and SDC/LCF8 composites exhibit the maximum power densities as 472 and 91 mW cm^−2^, respectively. For the SDC/LCF2 composite, a comparable OCV of 0.95 V is obtained. However, the OCV only reaches 0.66 V for the device using SDC/LCF8 composite. Therefore, it can be concluded that both SDC/LCF2 and SDC/LCF composites can yield good fuel cell performance. However, the majority of introducing Fe in the layered LCF8 material is an unfavorable factor in the SBFC technology.

As reported, the core component of SBFCs generally consists of two-part contributions: ionic conduction and electronic assisting transportation. In particular, the balance between the two parts is determinable to obtain good fuel cell performances. To investigate the optimum SDC/LCF composite for SBFC, we tailored the different weight ratios of SDC and LCF with 6:4, 7:3, and 8:2 (denoted as 6SDC/4LCF, 7SDC/3LCF, and 8SDC/2LCF). The fuel cell performances are displayed in Figure 5b. It can be observed that three of the devices show considerable OCVs of 1.07, 1.02, and 0.87 V and corresponding power densities of 544, 350, and 159 mW cm^−2^ for 6SDC/4LCF, 7SDC/3LCF, and 8SDC/2LCF, respectively. It indicates that the 6SDC/4LCF composite is the optimum one for SBFC application among the designed LCF series composites. The relatively electronic and ionic balance can be speculated in 6SDC/4LCF composite. However, the balance is likely to be broken when more ionic conducting SDC is introduced and negatively affects the fuel cell performance.

**Figure 6:**
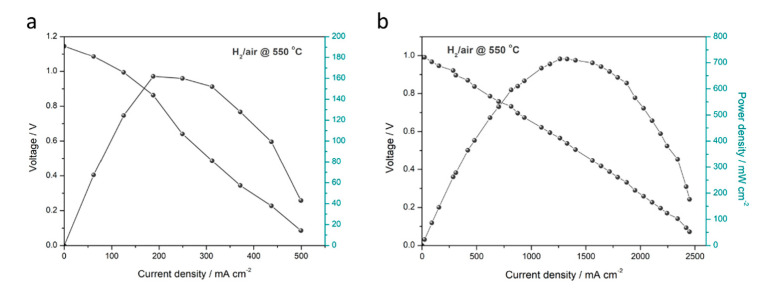
I–V and I–P characteristic of (**a**) the conventional SOFC using LCF as cathode and (**b**) the SBFC device using LCF as semiconductor membrane layer.

Furthermore, we explored the functionalities of the LCF as the cathode in conventional SOFC and core component in SBFC technology. As a typical mixed electronic and ionic conductivity material, LCF is considered to function as a cathode in a conventional fuel cell device with SDC as an electrolyte. The I–V and I–P characteristics of this fuel cell pellet operated at 550 °C are presented in Figure 6a. As observed, a high OCV of approximately 1.1 V is achieved, and the maximum power density reaches up to 162 mW cm^−2^. The considerable OCV indicates that LCF has a good catalytic property for oxygen reduction reaction (ORR) in the oxygen-input region. However, a relatively low current density is recorded. This can be attributed to the low electronic conductivity of LCF as cathode. Compared to the conventional device, the SBFC with a simplified configuration (Cell III) exhibits a comparable OCV of around 1 V, and the corresponding peak power density is 714 mW cm^−2^ (see Figure 6b). The significantly enhanced fuel cell performance (4.4-fold improved power density over that of conventional fuel cell device) profits from the SBFC and LCF can function as a core semiconducting ionic-conducting component in this technology. These findings expand the layer-structured LCF heterostructure composites into both conventional SOFC and recent-emerging SBFC technology.

## 4. Conclusions

In this study, a layer-structured LiCoO_2_–LiFeO_2_ composite is synthesized via a facile template-free hydrothermal method to use in low-temperature solid oxide fuel cells (SOFCs). Various molar ratios of LiCoO_2_–LiFeO_2_ are designed and investigated. The nominal composition LiCoO_2_-LiFeO_2_ with a molar ratio of 5:5 is proved as the optimum one. Based on the LiCoO_2_–LiFeO_2_ composite, the conventional SOFC and SBFC devices are fabricated. Additionally, the SBFC yields an enhanced power density output of 714 mW cm^−2^ at 550 °C, which is approximately 4.4 times over than that of the conventional SOFC (162 mW cm^−2^). The enhanced fuel cell performance is explained based on the high electrical conducting LiCoO_2_–LiFeO_2_ composite with multi-ion (e.g., H^+^ or/and O^2−^, even e^-^) conducting property. This work verifies a promising alternative to develop layered oxide semiconductor composites for high-performance LT-SOFC technology.

## Data Availability

Data is contained within the article or Supplementary Material.

## Supplementary Materials

The following are available online at https://www.mdpi.com/article/10.3390/nano11051224/s1, Figure S1: Diagram of SOFC testing station; Figure S2: EDS and composition (inset) of LCF sample.
