# Supplementary material for: Layered LiCoO2–LiFeO2 Heterostructure Composite for Semiconductor-Based Fuel Cells
Source: Nanomaterials (Basel). 2021 May 6;11(5):1224. doi: 10.3390/nano11051224 (PMC8148518; doi:10.3390/nano11051224)
Supplement: Supplementary file 1 [file nanomaterials-11-01224-s001.zip › nanomaterials-1179934-supplementary.pdf]

Layered $LiCoO_2$-$LiFeO_2$ Heterostructure Composite for Semiconductor-Based Fuel Cells

Yanyan Liu [1,*], Chen Xia [2], Baoyuan Wang [2,*] and Yongfu Tang [1]

1 Hebei Key Laboratory of Applied Chemistry, College of Environmental and Chemical Engineering, Yanshan University, Qinhuangdao, 064004, China
2 Faculty of Physics and Electronic Science, Hubei University, Wuhan, Hubei 430062, China
* Correspondence: Y.Y. Liu liuyy@ysu.edu.cn; B.Y. Wang baoyuanw@163.com

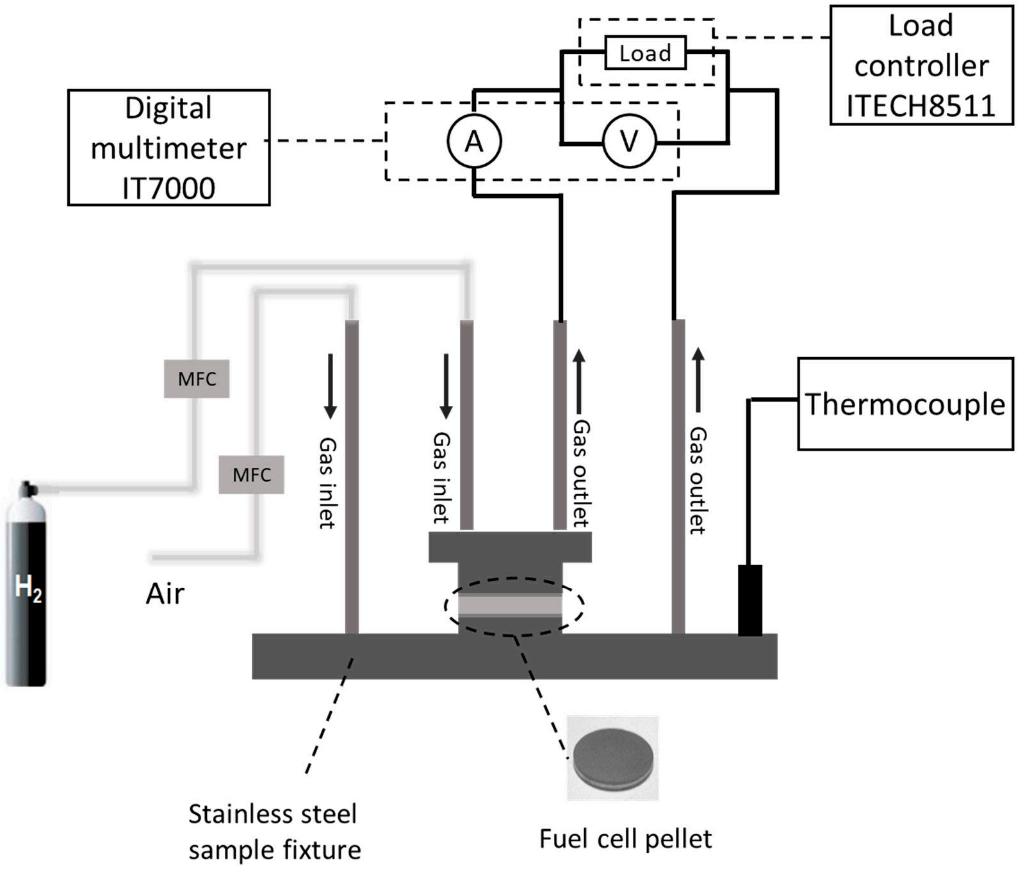

Figure S1. Diagram of SOFC testing station.

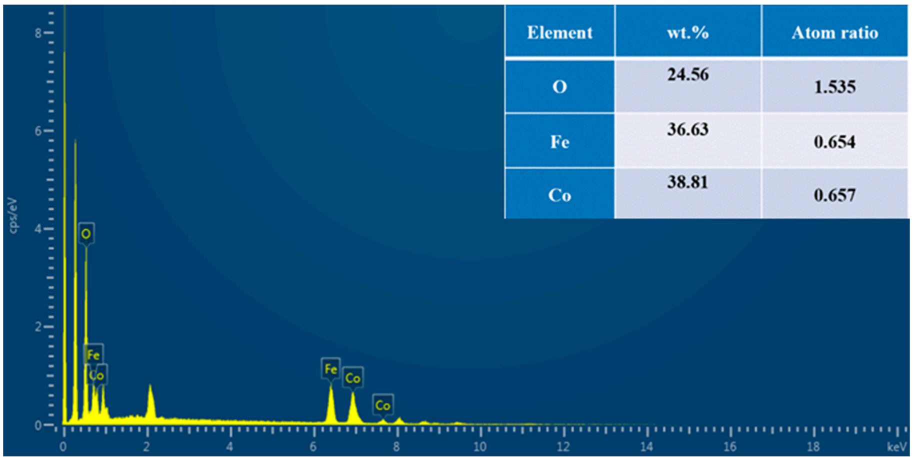

Figure S2. EDS and composition (inset) of LCF sample.
